# Association of maternal diet, micronutrient status, and milk volume with milk micronutrient concentrations in Indonesian mothers at 2 and 5 months postpartum

**DOI:** 10.1093/ajcn/nqaa200

**Published:** 2020-08-25

**Authors:** Rosalind S Gibson, Sofa Rahmannia, Aly Diana, Claudia Leong, Jillian J Haszard, Daniela Hampel, Malcolm Reid, Juergen Erhardt, Aghnia Husnayiani Suryanto, Wina Nur Sofiah, Annisha Fathonah, Setareh Shahab-Ferdows, Lindsay H Allen, Lisa A Houghton

**Affiliations:** Department of Human Nutrition, University of Otago, Dunedin, New Zealand; Faculty of Medicine, Universitas Padjadjaran, West Java, Indonesia; Department of Human Nutrition, University of Otago, Dunedin, New Zealand; Faculty of Medicine, Universitas Padjadjaran, West Java, Indonesia; Department of Human Nutrition, University of Otago, Dunedin, New Zealand; Department of Human Nutrition, University of Otago, Dunedin, New Zealand; USDA/ARS Western Human Nutrition Research Center, Davis, CA, USA; Department of Nutrition, University of California, Davis, CA, USA; Department of Chemistry, University of Otago, Dunedin, New Zealand; VitMin Lab, Willstaett, Germany; Faculty of Medicine, Universitas Padjadjaran, West Java, Indonesia; Faculty of Medicine, Universitas Padjadjaran, West Java, Indonesia; Faculty of Medicine, Universitas Padjadjaran, West Java, Indonesia; USDA/ARS Western Human Nutrition Research Center, Davis, CA, USA; Department of Nutrition, University of California, Davis, CA, USA; USDA/ARS Western Human Nutrition Research Center, Davis, CA, USA; Department of Nutrition, University of California, Davis, CA, USA; Department of Human Nutrition, University of Otago, Dunedin, New Zealand

**Keywords:** human milk micronutrient concentrations, human milk volume, maternal micronutrient intakes, maternal micronutrient deficits, maternal biomarkers, temporal changes in human milk nutrient concentrations

## Abstract

**Background:**

Maternal micronutrient deficits during preconception and pregnancy may persist during lactation and compromise human milk composition.

**Objective:**

We measured micronutrient concentrations in human milk and investigated their association with maternal micronutrient intakes, status, and milk volume.

**Methods:**

Infant milk intake (measured via a deuterium dose-to-mother technique), milk micronutrient and fat concentrations, and maternal micronutrient intakes were assessed at 2 and 5 mo postpartum in 212 Indonesian lactating mother–infant pairs. Maternal hemoglobin, ferritin, transferrin receptors, retinol binding protein (RBP), zinc, selenium, and vitamin B-12 were measured at 5 mo (*n* = 163). Multivariate or mixed effects regression examined associations of milk micronutrient concentrations with maternal micronutrient intakes, status, and milk volume.

**Results:**

Prevalence of anemia (15%), and iron (15% based on body iron), selenium (2.5%), and vitamin B-12 deficiency (0%) were low compared with deficiencies of zinc (60%) and vitamin A (34%). The prevalence of inadequate intakes was >50% for 7 micronutrients at 2 and 5 mo. Median milk concentrations for most micronutrients were below reference values, and nearly all declined between 2 and 5 mo postpartum and were not associated substantially with milk volume (except for β-carotene, α-carotene, and β-cryptoxanthin). At 5 mo postpartum, associations between maternal micronutrient status and corresponding milk concentrations reported as mean percentage difference in human milk concentration for each unit higher maternal biomarker were significant for hemoglobin (1.9%), iron biomarkers (ranging from 0.4 to 7%), RBP (35%), selenium (70%), and vitamin B-12 (0.1%), yet for maternal intakes only a positive association with β-carotene existed.

**Conclusions:**

Most milk micronutrient concentrations declined during lactation, independent of changes in human milk production, and few were associated with maternal micronutrient intakes. The significant associations between maternal biomarkers and milk micronutrient concentrations at 5 mo warrant further study to investigate whether the declines in milk micronutrients are linked to shifts in maternal status.

## Introduction

Multiple factors may cause growth faltering during infancy and early childhood in low- and middle-income countries (1), many of which have been associated with maternal micronutrient (MN) deficits during preconception and pregnancy (2–4). Major causes of these maternal MN deficits include inadequate dietary intakes, impaired absorption, and infection (3–5), all of which may persist during lactation (4, 6) and affect human milk composition (7).

Human milk concentrations of growth-limiting MNs (e.g., potassium, magnesium, phosphorus, zinc) are assumed to be unaffected by these maternal deficits, whereas low concentrations of vitamins A, D, E, C, and B; selenium; and iodine in human milk have been reported (8, 9). Nonetheless, whether these associations reflect more recent inadequate intakes during lactation or long-standing maternal MN deficiencies is often uncertain.

Human milk MN concentrations are impacted by prematurity (10) and fluctuate within feeds, diurnally, and with the stage of lactation (7, 11). Lack of standardized methods for the subsequent handling, storage, and analysis of human milk MNs are additional complicating factors (10, 11). Moreover, the influence of volume of human milk consumed by the infant on milk MN composition is uncertain, yet potentially important given the variations in milk production associated with exclusive and partial breastfeeding.

Recently, in a cross-sectional study, we investigated the association between maternal dietary intakes and human milk MN composition in a group of exclusively breastfed infants and their mothers. Despite substantial inadequacies in maternal intakes relative to the estimated average requirements of >40% for calcium, niacin, and vitamins A, B-6, and B-12, only a few positive associations existed between maternal nutrient intakes and MN concentrations in milk (12). However, assessment of maternal biomarker status was not conducted.

In light of these uncertainties, we applied state-of-the-art methods for collecting and measuring the human milk volume and MN quality, and assessed both maternal intakes and MN status in a cohort of disadvantaged breastfeeding Indonesian women. Our primary objective was to investigate associations between maternal MN status and human milk volume with MN concentrations at 5 mo postpartum. We also characterized the temporal changes in human milk MN concentrations from 2 to 5 mo postpartum and evaluated whether changes over time differed on the basis of the volume of milk produced.

## Methods

### Study site and participants

This study was part of a longitudinal field evaluation study designed to validate infant feeding practices and human milk intake using a shortened deuterium oxide dose-to-mother (DTM) technique. The study was conducted in 2 locations, the Bandung municipality and the Sumedang district, West Java, Indonesia, from June 2017 to January 2018. Urban participants resided in 1 of the most populated slum subdistricts of Bandung municipality, which has a population of 2.5 million. Details of the Sumedang district rural site have been reported previously (13). Both urban (*n* = 108) and rural (*n* = 104) lactating mothers were purposively recruited from local community health centers by health workers (cadres). Study participants who met inclusion criteria were women with no evidence of chronic disease or acute malnutrition who gave birth to a full-term infant (>37-wk gestation) with a birth weight of ≥2500 g and were currently breastfeeding. Measurements (except biomarkers) were recorded in the lactating mothers at both 2 and 5 mo (±2 wk) postpartum. The sample size of the study cohort was based on the overall aim of the validation study to distinguish exclusively breastfed (EBF) infants from non–exclusively breastfed infants [partially breastfed (PBF)] in the first 6 mo of life (14). After allowance for participant dropout, it was determined that 100 mother–infant pairs would be required in each group (EBF and PBF) to achieve this aim. For the current investigation, this sample size is sufficient to generate reliable estimates from regression models with several covariates (15).

Ethical approval was obtained from the Human Research Ethics Committee, Faculty of Medicine, Universitas Padjadjaran, Bandung, Indonesia (05/UN6.C1.3.2/KEPK/PN/2017). Informed written consent was obtained from all participants, who were free to leave the study at any time.

### Maternal sociodemographic and anthropometric status

Details on maternal sociodemographic status, parity, and time since last pregnancy were obtained using an interviewer-administered pretested questionnaire at 2 mo postpartum. Maternal weight was measured at both 2 and 5 mo postpartum and maternal height at 5 mo postpartum using calibrated equipment and standardized techniques (12) from which maternal BMI (kg/m^2^) was calculated.

### Laboratory assessment of maternal MN status

Maternal anticoagulated whole blood and serum were collected at 5 mo postpartum by trained phlebotomists from morning, nonfasting, venipuncture blood samples using rigorous trace element–free collection and separation procedures, as reported previously (16). Presence of symptoms of infection, time of blood collection, and time elapsed since last meal were all recorded. Blood was centrifuged (10 min at 2500 × *g*, 23°C) and serum was frozen on the day of collection and stored at −20°C until analysis.

Hemoglobin was assayed by means of a complete blood count using an automated counter (Sysmex XN-1000, Sysmex Corporation). Ferritin, serum transferrin receptor (sTfR), retinol binding protein (RBP), C-reactive protein (CRP), and α-1-acid-glycoprotein (AGP) were analyzed by a combined sandwich ELISA method (16). The interassay CVs were 2.3% for ferritin, 3.6% for sTfR, 3.6% for RBP, 5.8% for CRP, and 8.1% for AGP. Serum vitamin B-12 was analyzed by an automated electrochemiluminescence immunoassay (CV: 4.0%) using a commercial kit (vitamin B-12 Elecsys reagent kit, Roche Diagnostics, GmbH). Accuracy was checked via manufacturer controls and values fell within certified ranges. Serum zinc and selenium were determined by inductively coupled plasma mass spectrometry (ICP-MS) as described previously (16). An external control (UTAK, Utak Laboratories Inc., *n* = 24) was used to validate the accuracy, with the average serum zinc being 97% of the target with 1.5% CV, and the average serum selenium being 99% of target with 1.9% CV. The pooled samples were further matrix matched for precision checks, using low (*n* = 30) and medium (*n* = 21) pooled samples to achieve CVs of <2% for serum zinc and <3% for serum selenium.

Serum concentrations of ferritin, sTfR, RBP, zinc, and selenium were adjusted for inflammation using both CRP and AGP and the BRINDA (Biomarkers Reflecting Inflammation and Nutrition Determinants of Anemia) regression approach (17). Adjustments for serum zinc for time of day and time since last meal before the blood collection were made before adjusting for inflammation (18). Maternal body iron stores (BIS) were calculated using the ratio of sTfR and ferritin concentrations (adjusted for inflammation) according to the formula of Cook et al. (19). The cutoffs used to define deficiency for each biomarker were as follows: hemoglobin: <120 g/L; adjusted ferritin: <15 μg/L (20, 21); adjusted BIS: <0 mg/kg (19); adjusted sTfR: >8.3 mg/L (16); adjusted zinc: <10.7 μmol/L (22); adjusted RBP: <1.20 μmol/L (23); adjusted selenium: <0.82 μmol/L (16); and vitamin B-12 <148 pmol/L (24).

### Assessment of maternal MN intakes

Food intakes at 2 and 5 mo postpartum were assessed from in-home weighed food records on 3 nonconsecutive days. In Bandung city, participants were trained to weigh their own food intakes, whereas in Sumedang district, trained community cadres weighed 12-h food intakes (06:00–18:00) in the home and performed 12-h maternal recalls of any foods consumed over the previous 18:00–06:00 period to estimate the total 24-h food intake (6). Maternal nutrient intakes were calculated using a locally produced Indonesian food composition table that took into account the government mandatory fortification of all wheat flour products with 5 MNs (iron: 50 mg/kg; zinc: 30 mg/kg; thiamin: 2.5 mg/kg; riboflavin: 4 mg/kg; folate, 2 mg/kg) (6). To assess the adequacy of the MN intakes, the estimated average requirements (EARs) for lactating women compiled by the WHO as modified by Arimond et al. (25) were used except for zinc, calcium, iron, and potassium. For zinc, the EARs from the International Zinc Nutrition Consultative Group (IZiNCG) for mixed refined vegetarian diets was used, whereas for calcium and iron the EARs from the Institute of Medicine (IOM) were used, with the iron adjusted to account for 10% bioavailability as described previously (12). For potassium, only an adequate intake (AI) is available (26) so the prevalence of inadequacy could not be assessed.

### Collection and MN analysis of human milk samples

Full human milk samples were collected in the morning on day 14 (after deuterium oxide dosing) at both 2 and 5 mo postpartum using a breast pump (Harmony, Medela), after the mothers were instructed to take strict precautions to avoid all sources of adventitious contamination as described previously (12). A full human milk sample from the selected breast was transferred into an acid-washed trace element–free glass bottle. After gentle mixing, aliquots (1 mL) of human milk were transferred into acid-washed microtubes covered in aluminum foil to minimize degradation of photosensitive vitamins, frozen on the day of collection, and stored at −80°C until analysis.

Milk fat concentration was measured in fresh milk via the creamatocrit method (CreamatocritPlus; EKF Diagnostics) (27) (CV: 6.9%) to express human milk retinol and vitamin E per g fat as well as per unit volume (12). Analysis of the minerals (sodium, magnesium, phosphorus, potassium, calcium, iron, copper, zinc, and selenium) in human milk were carried out in the Centre for Trace Element Analysis, Department of Chemistry, University of Otago by ICP-MS (Agilent 7900, Agilent Technologies), as previously described (12). Precision and accuracy of the elements were checked against in-house pooled samples and a multielement reference standard (SRM 1846, infant formula) from the National Institute of Standards and Technology.

Analysis of the vitamins was conducted at the USDA Agricultural Research Service Western Human Nutrition Research Center (WHNRC). Briefly, free thiamin, thiamin monophosphate (TMP), and thiamin pyrophosphate (TPP) (CV range from 5.9% to 11.3%) were analyzed by HPLC with fluorescence detection, performed with an Agilent 1200 series HPLC by precolumn derivatization into the thiochrome esterase, as previously described (28) with few modifications: only 100 µL whole milk was used for analysis, and 4-deoxypyridoxine was added as an internal standard to account for volume changes. Total thiamin was calculated based on the measured concentrations for each vitamer and expressed as free thiamin [thiamin + (TPP × 0.707) + (TMP × 0.871)]. Riboflavin, FAD, FMN, nicotinamide, NAD, pantothenic acid, pyridoxal (PL), pyridoxine (PN), and biotin (CV range from 7.5% to 12.7%) were analyzed in multiple reaction monitoring mode by ultra-performance liquid chromatography (UPLC) MS/MS (29) with a Waters ACQUITY UPLC I-class system coupled to a Sciex 4500 triple quadrupole mass spectrometer. Total riboflavin was calculated based on the measured concentrations for each vitamer and expressed as riboflavin [riboflavin + (FAD × 0.479) + (FMN × 0.825)]. Niacin was calculated as nicotinamide [nicotinamide + (NAD × 0.184)]. Vitamin B-6 was calculated as [PL + PN]. Vitamin B-12 (cobalamin) (CV: 10.7%) was analyzed by competitive chemiluminescent enzyme immunoassay (IMMULITE 1000; Siemens) as previously described (30). Preformed retinol and 3 provitamin A carotenoids (α-carotene, β-carotene, and β-cryptoxanthin), α-tocopherol, and γ-tocopherol (CV range from 4.9% to 19.2%) were determined by an Agilent 1260 HPLC system with multiwavelength detection as previously described (11). Vitamin E was expressed as tocopherol equivalents (TE) defined as α-tocopherol + 0.25 γ-tocopherol (10).

### Assessment of human milk volume

Intake of human milk (mL/d) was derived from data generated by the DTM technique over 14 d. Predose saliva samples were collected on day 0 from both mother and infant, after which mothers were given an accurately measured oral dose of diluted deuterium oxide as described earlier (12). Next, instead of the International Atomic Energy Agency full DTM protocol requiring 7 postdose saliva samples collected from the mothers and infant over a 14-d study period (12), a 2-postdose saliva collection design was used whereby samples were collected from the mother and infant on days 7, 8, or 9, and days 13 or 14 as validated and described by Liu et al. (14). Care was taken to ensure that neither mother nor infant had consumed food or fluid at least 30 min or 15 min, respectively, prior to each saliva collection. All saliva samples were frozen on the day of collection and stored at −20°C until analysis of deuterium enrichment by Fourier transform infrared spectrometry (Agilent 4500). A fully Bayesian framework using a gradient-based Markov chain Monte Carlo approach implemented in Stan (14) was used to calculate the average amount of human milk intake over a 14-d period.

### Statistical analysis

All statistical analysis was carried out using Stata 16 (Stata Corp. 2019, Stata Statistical Software: Release 16, Stata Corp LLC). Only mothers who had either dietary data at both 2 and 5 mo postpartum, or MN status (biomarkers) and milk composition data at 5 mo postpartum, or milk composition data at both 2 and 5 mo postpartum were included.

Dietary intake was described using medians and 25th and 75th percentiles at both timepoints. Prevalence of inadequate intakes was determined by applying the probability approach to the usual intake distributions (31). A probability risk curve for iron for lactating women was not available, so this curve was generated based on 10% bioavailability using an EAR of 11.7 mg/d (lactating adults) and 12.6 mg/d (lactating adolescents) and CV of 30% (25). Differences in dietary intakes between 2 and 5 mo postpartum were calculated for each participant and mean differences and 95% CIs reported.

To describe the concentration of MNs in human milk at 2 and 5 mo postpartum, medians and 25th and 75th percentiles were reported separately for each time and also pooled, by first calculating the within-person mean concentrations from 2 and 5 mo. Estimation of the association between human milk volume and human milk MN concentrations was made using mixed-effects regression models, which combined data from 2 and 5 mo postpartum. Participant was included as a random effect and the estimates were adjusted for parity. Human milk MN concentrations, as the outcome variables, were first log-transformed to correct skew, and were estimates back-transformed to be presented as percentage change. To check whether the relations were different at 2 or 5 mo postpartum, the model was run with an interaction term between time and milk volume. If the interaction term had a *P* value < 0.1, then associations were recalculated stratified by time.

To investigate whether changes in milk volume were associated with changes in MN concentrations from 2 to 5 mo postpartum, the sample was first categorized into the following 2 groups: those who had a decrease in milk volume by >100 mL between 2 and 5 mo postpartum and those whose milk volume did not decrease by >100 mL. This dichotomization was carried out to facilitate meaningful interpretation of the results and to reduce the possibility of spurious associations with small changes in milk volume over the 3 mo. Mean changes (95% CI) in MN concentrations were calculated for each group. The mean difference and 95% CI for the changes of MN concentrations in human milk between groups was also calculated.

Associations between maternal biomarkers and human milk MN concentrations were estimated using linear regression models. Human milk MN concentrations, as the outcome variables, were log-transformed as before. All analyses were adjusted for human milk volume, parity, urban or rural setting, and inflammation (AGP).

To determine the cross-sectional association between maternal dietary intake of MNs and milk concentration of the same MN (or a fraction of it), linear regression models were used as described previously, and all models were adjusted for milk volume and parity (**Supplemental Table 1**).

Residuals of all regression models were plotted and visually assessed for homogeneity of variance and normality. Emphasis on estimation over statistical testing has been used in this analysis, with limited reliance on *P* values for inference, as recommended (32).

## Results

Of the 221 participants, 212 had longitudinal data available for either milk MN concentrations or maternal dietary intakes at both 2 and 5 mo postpartum; of these participants, 163 had data available for maternal MN status and milk MN concentrations at 5 mo postpartum (**Supplemental Figure 1**). Mothers who were excluded were not different from those who were not in terms of age, infant sex, parity, height, and BMI; however, those excluded from the human milk MN composition and dietary intake analyses (*n* = 9) were, on average, more wealthy (mean wealth score 0.6 compared with 0.0), and less likely to live in a rural setting (33% compared with 49%), whereas mothers excluded from the MN status and milk MN composition analyses (*n* = 49) were more likely to have a junior high school education (49% compared with 30%) and less likely to have an elementary school education (14% compared with 21%) or a senior high school education (37% compared with 48%).

Characteristics of the 212 mothers are presented in **Table 1**. The mean maternal age was 28 y, fewer than one-half of the mothers had completed senior high school and above, and one-third were primiparous. The mean birthweight of the infants was 3126 g, and 49% (104 of 212) were female. At 5 mo postpartum, mean maternal BMI was 24.3, with more than one-third of the mothers classified as overweight or obese. The only dietary supplements that mothers consumed were vitamin A capsules (59%; 89 of 152) during the early postpartum period (≤40 d postpartum).

**TABLE 1:** Demographic characteristics and biomarkers of mothers (*n* = 212)^1^

Characteristic	Value
Age at 5 mo postpartum, y	28.4 ± 6.4
Wealth index^2^	0.0 ± 1.3
Education	
Elementary school (6–7 y old/6-y duration)	42 (19.8)
Junior high school (12–13 y old/3-y duration)	73 (34.4)
Senior high school (15–16 y old/≥3-y duration)	97 (45.8)
Rural location	104 (49.1)
Infant sex female	104 (49.1)
Infant birthweight, g	3126 ± 344
Primiparous	70 (33.0)
Time since last pregnancy,^3^ mo	45 (0, 80)
Height at 5 mo postpartum, cm	151 ± 5
Maternal height <145 cm	28 (13.2)
BMI at 5 mo postpartum	24.3 ± 3.9
Weight status at 5 mo postpartum^4^	
Underweight	9 (4.2)
Overweight	68 (32.1)
Obese	16 (7.6)
Human milk volume	
2 mo postpartum (83% EBF)	746 ± 214
5 mo postpartum (43% EBF)	701 ± 187
Biomarkers at 5 mo postpartum^5^ (*n* = 163)	
Hemoglobin, g/L	132 ± 12
Hemoglobin <120 g/L	25 (15.3)
Iron serum ferritin, geometric mean (95% CI) μg/L	24.9 (21.5, 28.8)
Iron serum ferritin <15 μg/L	40 (24.5)
Iron sTfR, geometric mean (95% CI) mg/L	6.27 (5.99, 6.57)
Iron sTfR >8.3 mg/L	26 (16.0)
Iron BIS, mg/kg	4.36 ± 4.20
Iron BIS <0 mg/kg	25 (15.3)
IDA (hemoglobin <120 g/L and BIS <0 mg/kg)	12 (7.4)
Zinc, μmol/L	10.4 ± 1.3
Zinc <10.7 μmol/L	98 (60.1)
RBP, geometric mean (95% CI) μmol/L	1.33 (1.28, 1.39)
RBP <1.20 μmol/L	56 (34.4)
Selenium, μmol/L	1.10 ± 0.16
Selenium <0.82 μmol/L	4 (2.5)
Selenium <0.89 μmol/L	12 (7.4)
Selenium <1.0 μmol/L	35 (21.5)
Selenium <1.1 μmol/L	85 (52.2)
Vitamin B-12, pmol/L	301 ± 117
Vitamin B-12 <148 pmol/L	0
CRP, median (25th, 75th percentiles) mg/L	1.14 (0.45, 2.54)
CRP >5 mg/L	24 (14.7)
AGP, median (25th, 75th percentiles) g/L	0.83 (0.63, 1.07)
AGP >1 g/L	53 (32.5)

1 Values are means ± SDs, number of participants (%), or median (25th, 75th percentile) unless otherwise indicated. AGP, α-1-acid-glycoprotein; BIS, body iron stores; CRP, C-reactive protein; EBF, exclusively breastfed; IDA, iron deficiency anemia; RBP, retinol binding protein; sTfR, serum transferrin receptor.

2 An asset-based wealth index derived following the Demographic Health Survey Wealth Index guidelines (33), from principal component analysis.

3 Pregnancy distance between the previous child and the infant participant in the study, when child was born.

4 Underweight defined as BMI <18.5; overweight as BMI ≥25 and <30; obese defined as BMI ≥30.

5 Cutoffs used to define deficiency for each biomarker were as follows: hemoglobin, <120 g/L; adjusted ferritin, <15 μg/L (20, 21); adjusted BIS, <0 mg/kg (19); adjusted sTfR, >8.3 mg/L (16); adjusted zinc, <10.7 μmol/L (22); adjusted RBP, <1.20 μmol/L (23); adjusted selenium, <0.82 μmol/L (16), <0.89, <1.0, <1.1 μmol/L (34); vitamin B-12, <148 pmol/L (24). Zinc adjusted for time of the day and interval since the last meal = exp (unadjusted in biomarkers) + regression coefficient for time of day × [(time of day − regression coefficient for interval since previous meal) × interval since previous meal]. Ferritin, sTfR, RBP, zinc, and selenium adjusted for inflammation = exp (unadjusted in biomarkers − regression coefficient for CRP) × (CRP − maximum of lowest decile for CRP) – [regression coefficient for AGP × (AGP − maximum of lowest decile for AGP)].

The prevalence of iron deficiency was 25% based on low serum ferritin concentrations, 16% based on elevated concentrations of sTfR, and 15% based on low body iron. Anemia was present in 15% of mothers, of whom 7% had iron deficiency anemia based on hemoglobin <120 g/L and adjusted BIS <0 mg/kg. The prevalence of zinc and RBP deficiency was high (60% and 34%, respectively), whereas that for both selenium and vitamin B-12 deficiency was low (2.5% and 0%, respectively), albeit the prevalence of selenium deficiency varied based on the cutoff employed (Table 1). The prevalence of elevated CRP and AGP, biomarkers of inflammation and infection, were 15% and 33%, respectively. In addition, rural women had higher hemoglobin, RBP, and AGP concentrations as well as lower zinc and selenium concentrations compared with urban women, in addition to being significantly younger, less wealthy, less educated, and more likely to be overweight (all *P* < 0.05; data not shown).

Median (25th, 75th percentiles) usual daily MN intakes of the mothers (*n* = 210) at 2 and 5 mo postpartum from dietary sources alone are shown in **Table 2**. Two mothers were excluded from these analyses because dietary intake data were missing at either 2 or 5 mo postpartum. Changes in MN intake between 2 and 5 mo postpartum were small. The prevalence of inadequate intakes was >50% for 7 MNs at both 2 and 5 mo postpartum, including all of the assessed B-vitamin intakes, even though thiamin and riboflavin are included as fortificants in wheat flour products. The prevalence of inadequate intake of iron, also targeted by the national wheat fortification program, was close to 50% at 2 mo (46%) and 5 mo postpartum (47%). The median (25th, 75th percentiles) intake for potassium at both time points fell well below the AI set by the IOM (26). Only zinc had a prevalence of inadequacy of <25% at both 2 and 5 mo postpartum.

**Table 3** presents the median (25th, 75th percentiles) concentrations of 18 MNs in human milk together with the individual vitamers for provitamin A carotenoids, vitamin E, thiamin, riboflavin, niacin, and vitamin B-6, pooled at 2 and 5 mo postpartum. Nineteen mothers were excluded here due to missing milk composition data at either 2 or 5 mo postpartum. Compared with mothers with full data for this analysis, excluded mothers were older (mean age 31.2 y compared with 28.2 y), more wealthy (mean wealth score 0.6 compared with 0.0), more educated (72% with senior high school compared with 43%), less likely to be rural (6% compared with 53%), more likely to have a male infant (67% compared with 48%), and to be of higher BMI (mean 26.0 kg/m^2^ compared with 24.2 kg/m^2^). Corresponding MN concentrations for mature human milk compiled by the European Food Safety Authority (EFSA) (35–44) and IZiNCG (45) are shown for comparison. The majority of milk minerals and vitamins analyzed, when pooled at 2 and 5 mo postpartum, were below the range of the EFSA values, especially the B vitamins, which were concerningly low, with the exception of biotin (B-7).

Human milk MN concentrations were not affected substantially by milk volume (i.e., small effect size), with the exception of β-carotene, α-carotene, and β-cryptoxanthin, which increased between 8% and 10% for each 100 mL of greater milk volume output (Table 3). Interactions were indicated between time and milk volume in predicting MN milk concentrations for calcium, selenium, TMP, riboflavin and FMN—all of which were more strongly related to milk volume at 5 mo postpartum; in contrast, zinc was more strongly related at 2 mo postpartum.

Temporal declines in most milk MN concentrations were observed between 2 and 5 mo postpartum (**Table 4**) regardless of whether participants did or did not decrease their milk volume by 100 mL; although potassium declined more substantially from 2 to 5 mo postpartum in those participants whose milk volume did not compared with those whose milk volume did decrease by ≥100 mL between these 2 time periods.

Concentrations of the 3 maternal iron biomarkers and hemoglobin were associated with milk iron concentrations at 5 mo postpartum after adjustment for parity, human milk volume, rural living status, and inflammation (AGP) (**Table 5**). Positive associations between maternal RBP, selenium, and vitamin B-12 concentrations in serum and milk concentrations of retinol and β-cryptoxanthin, selenium, and vitamin B-12, respectively, were also apparent. For each unit higher for each maternal biomarker, corresponding increases in milk concentrations ranged from 0.4% (95% CI: 0.1%, 0.7%) for ferritin to 66% (95% CI: 20%, 130%) for selenium.

**TABLE 2:** Maternal daily MN intakes at 2 and 5 mo postpartum and prevalence of inadequacy (*n* = 210)^1^

		2 mo postpartum		5 mo postpartum		
	EAR^2^	Median (25th, 75th percentiles)	Prevalence of inadequacy (%)^3^	Median (25th, 75th percentiles)	Prevalence of inadequacy (%)^3^	Mean difference (95% CI) between 2 and 5 mo
Calcium, mg	800^4^	497 (410, 604)	95.7	471 (376, 563)	97.3	−38 (−57, −18)
Potassium, g	5.1^5^	1.0 (0.8, 1.2)	—	0.9 (0.7, 1.1)	—	−0.03 (−0.08, 0.01)
Iron, mg	11.7^6^	11.8 (10.0, 14.7)	45.7	12.2 (9.3, 14.7)	46.6	−0.2 (−0.7, 0.3)
Zinc, mg	7^7^	9.3 (7.9, 11.3)	14.1	9.5 (7.4, 11.4)	21.7	−0.3 (−0.7, 0.1)
Vitamin A, μg RAE	450	398 (282, 565)	56.9	387 (298, 548)	58.4	−27 (−64, 10)
Thiamin (B-1), mg	1.2	1.0 (0.8, 1.2)	72.1	1.1 (0.8, 1.3)	62.2	0.05 (−0.004, 0.10)
Riboflavin (B-2), mg	1.3	1.2 (1.0, 1.4)	67.2	1.2 (0.9, 1.5)	60.6	0.02 (−0.03, 0.07)
Niacin (B-3), mg	13	10.5 (8.5, 13.1)	71.2	10.9 (8.7, 13.6)	67.9	0.5 (−0.1, 1.0)
Vitamin B-6, mg	1.7	1.1 (0.9, 1.3)	91.5	1.3 (1.0, 1.6)	77.2	0.2 (0.1, 0.3)
Vitamin B-12, μg	2.4	2.3 (1.7, 3.0)	54.9	2.1 (1.5, 3.2)	56.2	0.4 (−0.3, 1.0)

1 Mean difference was calculated using a *t*-test. Maternal MN intakes were based on food records on 3 nonconsecutive days (weighed 12-h daytime food intakes added with 12-h recalls), with adjustment for usual intakes using the multiple source method (46). AI, adequate intake; EAR, estimated average requirement; IOM, Institute of Medicine; IZiNCG, International Zinc Nutrition Consultative Group; MN, micronutrient; RAE, retinol activity equivalents.

2 EAR values were obtained from the WHO as modified by Arimond et al. (25), unless otherwise stated.

3 Using the probability method for assessing prevalence of inadequate intakes for all MNs.

4 Values were obtained from the 2011 updated IOM dietary reference values (47), and as the EARs for lactating women aged 14–18 y were higher (1100 mg), this EAR was used for these women (*n* = 9 at 2 mo; *n* = 8 at 5 mo).

5 No EAR value for potassium, therefore the AI is reported (26) and the prevalence of inadequacy cannot be determined.

6 Values adapted for 10% bioavailability of iron, EAR of 11.7 mg/d (lactating adults) and 12.6 mg/d (lactating adolescents) (25).

7 Value obtained from IZiNCG, assuming bioavailability of mixed refined vegetarian diets (48).

Associations between milk MN and maternal MN intakes were generally weak (**Supplemental Table 1**). Statistically significant (*P* < 0.05) associations existed at 2 mo postpartum between concentrations of calcium, iron, zinc, and niacin (as nicotinamide) in human milk and maternal intakes, which were positive for calcium, iron, and niacin but negative for zinc. At 5 mo postpartum, a significant and positive association was observed only between β-carotene human milk concentration and vitamin A intake. However, the effect sizes at both 2 and 5 mo postpartum were small, especially for calcium and vitamin A (both <1%).

## Discussion

In the present longitudinal study, breastfeeding mothers were both consuming suboptimal diets and at risk of multiple MN deficiencies, including iron, zinc, and vitamin A. Concentrations of most milk MNs were low in comparison with the most recent values for mature human milk compiled by EFSA (35–44), particularly at 5 mo postpartum. In general, a greater human milk volume was associated with higher concentrations of most MNs, most notably for the provitamin A carotenoids and fat-soluble vitamins (Table 3).

### Associations between maternal intakes and status and human milk concentrations

The study participants who were disadvantaged mothers had diets with substantial MN inadequacies, consistent with our previously reported findings (12), yet few associations existed between human milk MN concentrations and maternal intakes at 2 and 5 mo postpartum. For the few associations that did exist, the effect sizes were small, particularly for calcium and vitamin A (Supplemental Table 1). In contrast, we observed positive relations for human milk retinol, β-cryptoxanthin, vitamin B-12, selenium, and iron at 5 mo postpartum relative to the maternal biomarker status of the study participants, findings consistent with most reports (49–51) with the exception of those for iron (52, 53) (Table 5).

The positive association between human milk nicotinamide and maternal niacin intake is consistent with our previous findings (12). However, at 5 mo postpartum the associations we observed between human milk iron, maternal iron intake at 2 mo postpartum, and all of the maternal biomarkers of iron status we investigated (hemoglobin, ferritin, sTfR, and BIS) were unexpected, and to our knowledge similar findings have not been reported previously (52, 53). Whether these findings were attributable to our analysis of human milk iron via a more precise analytical method (ICP-MS) (54) than used previously is uncertain. Of the disadvantaged mothers who participated in the study, only a few (15%) had a low BIS at 5 mo postpartum, a finding that was likely a reflection of >50% study participants with adequate iron intakes coupled with maternal amenorrhea.

The reason for the negative relation noted here between maternal zinc intake and milk zinc concentrations at 2 mo postpartum is unclear. Several other investigators have reported a comparable inverse relation among women of low socioeconomic status (55–57). Contamination of milk samples at the time of collection is unlikely to be the explanation for this finding, because strict precautions were employed during the collection and analyses of the milk samples both here and in these earlier studies. Moreover, our milk zinc concentrations at 2 and 5 mo postpartum were comparable to the values compiled by EFSA (10) and IZiNCG (45). Elevated levels of stress-mediated carrier proteins, including α2-macroglobulin, reported in postpartum women of low socioeconomic status (58), such as the disadvantaged Indonesian women studied here, have been posited to increase mammary zinc secretion (59) and warrant investigation.

**TABLE 3:** Association between human milk MN concentration and human milk volume at 2 and 5 mo postpartum (*n* = 193)^1^

		Pooled human milk concentration at 2 and 5 mo			Mean % change (95% CI) for 100 mL greater milk volume at 2 mo postpartum^5^	
	Mature milk concentration reference^2^	Median (25th, 75th percentile)^3^	Mean % change (95% CI) for 100 mL greater milk volume^4^	*P* value for time–milk volume interaction^5^		Mean percentage change (95% CI) for 100 mL greater milk volume at 5 mo postpartum^5^
Sodium, mg/L	150 (35)	117 (98, 151)	−2.5 (−5.6, 0.7)			
Magnesium, mg/L	31 (35)	28 (25, 32)	−0.9 (−1.8, 0.1)			
Phosphorous, mg/L	140 (36)	133 (121, 146)	0.4 (−0.6, 1.5)			
Potassium, mg/L	500 (35)	438 (402, 475)	**−**0.8 (−1.6, −0.1)			
Calcium, mg/L	250 (35)	256 (233, 275)	0.7 (−0.1, 1.5)	0.020	−0.4 (−1.3, 0.4)	1.1 (−0.1, 2.2)
Iron, mg/L	0.3 (37)	0.22 (0.17, 0.29)	0.2 (−2.9, 3.4)			
Copper, mg/L	0.35 (35)	0.27 (0.22, 0.30)	1.4 (−0.3, 3.2)			
Zinc, mg/L	1.91 (1 to 2 mo)	1.1 (0.8, 1.4)	1.0 (−2.0, 4.1)	0.070	−3.1 (−6.5, 0.4)	−0.1 (−4.0, 4.0)
	0.98 (3 to 5 mo) (45)					
Selenium, μg/L	15 (38)	13.3 (11.8, 15.3)	−1.5 (−3.2, 0.2)	0.016	−0.8 (−2.7, 1.1)	−3.7 (−6.3, −1.2)
Retinol, μg/L	530 (39)	530 (399, 650)	3.3 (0.5, 6.1)			
Retinol^6^, μg/g milk fat		11.2 (8.6, 14.0)	1.3 (−1.1, 3.8)			
β-Carotene, μg/L		21 (14, 32)	10.3 (5.6, 15.2)			
α-Carotene, μg/L		9.6 (6.2, 12.6)	8.1 (4.0, 12.2)			
β-Cryptoxanthin, μg/L		26 (17, 43)	7.5 (2.6, 12.7)			
α-Tocopherol^6^, mg/L		5.0 (3.9, 6.4)	3.6 (0.9, 6.2)			
α-Tocopherol^6^, mg/g milk fat		0.11 (0.08, 0.14)	1.6 (−0.8, 4.0)			
γ-Tocopherol, mg/L		0.45 (0.33, 0.63)	4.3 (1.3, 7.3)			
γ-Tocopherol^6^, mg/g milk fat		0.010 (0.007, 0.013)	2.3 (−0.5, 5.1)			
Vitamin E (as TE)^6,7^, mg/L	4.6 (40)	5.2 (4.0, 6.5)	3.7 (1.1, 6.4)			
Vitamin E (as TE)^6,7^, mg/g milk fat		0.11 (0.09, 0.14)	1.6 (−0.7, 3.9)			
Free thiamin, μg/L		18.5 (14.2, 24.0)	−1.2 (−3.7, 1.3)			
TMP, μg/L		94 (74, 110)	4.2 (1.1, 7.2)	0.006	1.0 (−2.2, 4.3)	7.7 (2.7, 12.6)
TPP, μg/L		4.5 (3.2, 5.7)	−4.6 (−7.1, −2.0)			
Total thiamin (B-1),^8^ μg/L	180 (41)	104 (88, 121)	1.3 (-0.3, 3.0)			
Free riboflavin, μg/L		11.2 (7.4, 15.6)	5.8 (0.9, 10.9)	0.029	1.4 (−2.5, 5.3)	8.7 (0.2, 17.2)
FAD, μg/L		166 (137, 208)	−0.1 (−2.6, 2.4)			
FMN, μg/L		15.6 (9.4, 22.8)	0.2 (−3.6, 4.2)	0.021	−1.1 (−6.1, 3.9)	3.9 (−1.8, 9.7)
Total riboflavin (B-2),^9^ μg/L	364 (42)	108 (91, 138)	0.2 (−2.0, 2.4)			
Nicotinamide, μg/L		275 (189, 411)	**−**4.2 (−7.5, −0.7)			
NAD, μg/L		2493 (1567, 3733)	−3.5 (−7.3, 0.4)			
Total niacin (B-3),^10^ μg/L	2100 (43)	737 (551, 1120)	**−**4.0 (−7.1, −0.7)			
Pantothenic acid (B-5), μg/L	2500 (35)	1304 (1069, 1509)	−1.7 (−4.7, 1.5)			
Pyridoxal, μg/L		96 (69, 122)	1.1 (−1.8, 4.0)			
Vitamin B-6,^11^ μg/L	130 (35)	96 (69, 122)	1.1 (−1.7, 4.0)			
Biotin (B-7), μg/L	5 (35)	6.4 (4.7, 7.9)	−2.0 (−6.3, 2.4)			
Vitamin B-12, μg/L	0.5 (44)	0.29 (0.26, 0.35)	−0.7 (−3.2, 1.8)			

1 ESFA, European Food Safety Authority; IZiNCG, International Zinc Nutrition Consultative Group; MN, micronutrient; TE, tocopherol equivalent; TMP, thiamin monophosphate; TPP, thiamin pyrophosphate.

2 Corresponding MN concentration for mature human milk compiled by EFSA and IZiNCG.

3 Median of the mean concentrations at 2 and 5 mo postpartum.

4 Mixed-effects regression models were used that combined the repeat measure and included participant as a random effect as well as adjusted for parity.

5 Stratified associations of human milk volume and human milk MN concentrations by time for interactions with *P* < 0.1 from column “Mean % change (95% CI) for 100 mL greater milk volume.”

6 *n* = 1 missing milk fat–associated MN value at 2 mo; *n* = 1 missing α-tocopherol, *n* = 1 missing milk fat–associated MN value at 5 mo.

7 Vitamin E calculated as the sum of each vitamer expressed as TEs, where 1 mg α-tocopherol = 1 TE, 0.25 mg γ-tocopherol = 1 TE (10).

8 Vitamin B-1 was calculated based on the measured concentrations for each vitamer and expressed as free thiamin [thiamin + (TPP × 0.707) + (TMP × 0.871)].

9 Vitamin B-2 was calculated based on the measured concentrations for each vitamer and expressed as riboflavin [riboflavin + (FAD × 0.479) + (FMN × 0.825)].

10 Vitamin B-3 was calculated as nicotinamide [nicotinamide + (NAD × 0.184)].

11 Vitamin B-6 was calculated as (pyridoxal + pyridoxine).

**TABLE 4:** Change in human milk MN concentrations according to decrease in human milk volume between 2 and 5 mo postpartum (*n* = 193)^1^

	Median (25th, 75th percentiles) human milk concentration		Mean change (95% CI) in human milk concentration between 2 and 5 mo postpartum		Mean difference in change (95% CI) in human milk concentration between those who decreased their volume by >100 mL between 2 and 5 mo and those who did not^2^
	2 mo postpartum (*n* = 193)	5 mo postpartum (*n* = 193)	Participants who decreased their human milk volume by >100 mL (*n* = 62)	Participants who did not decrease their human milk volume by at least 100 mL (*n* = 131)	
Sodium, mg/L	123 (101, 173)	101 (83, 122)	−21 (−83, 41)	−14 (−54, 26)	7 (−65, 78)
Magnesium, mg/L	29 (25, 32)	28 (24, 32)	−0.2 (−1.9, 1.5)	−0.9 (−1.7, −0.2)	−0.7 (−2.3, 0.9)
Phosphorous, mg/L	138 (125, 155)	127 (115, 142)	**−**7 (−14, −1)	**−**13 (−18, −9)	−6 (−14, 2)
Potassium, mg/L	450 (417, 485)	420 (384, 462)	−9 (−25, 7)	−34 (−44, −24)	**−**25 (−43, −7)
Calcium, mg/L	267 (247, 288)	239 (220, 267)	**−**21 (−29, −13)	**−**29 (−33, −24)	−8 (−16, 1)
Iron, mg/L	0.26 (0.20, 0.33)	0.17 (0.13, 0.24)	−0.10 (−0.21, 0)	**−**0.13 (−0.17, −0.09)	−0.03 (0.12, 0.06)
Copper, mg/L	0.30 (0.25, 0.35)	0.23 (0.18, 0.27)	**−**0.06 (−0.08, −0.03)	**−**0.08 (−0.09, −0.06)	−0.02 (−0.05, 0.005)
Zinc, mg/L	1.3 (0.9, 1.8)	0.8 (0.6, 1.1)	**−**0.50 (−0.62, −0.39)	**−**0.51 (−0.59, −0.42)	−0.01 (−0.15, 0.14)
Selenium, μg/L	13.9 (12.3, 16.6)	12.2 (10.6, 14.7)	−0.75 (−4.1, 2.6)	**−**2.0 (−2.8, −1.1)	−1.2 (−3.8, 1.4)
Retinol, μg/L	644 (472, 857)	386 (279, 516)	**−**249 (−353, −145)	−333 (−404, −261)	−84 (−209, 42)
Retinol,^3^ μg/g milk fat	12.1 (9.5, 16.9)	9.3 (6.8, 12.6)	**−**3.6 (−5.6, −1.3)	**−**3.6 (−4.7, −2.4)	−0.1 (−2.3, 2.1)
β-Carotene, μg/L	30.1 (19.5, 44.4)	12.2 (7.6, 19.1)	**−**19 (−31, −8)	**−**24 (−28, −19)	−4 (−14, 5)
α-Carotene, μg/L	12.6 (8.2, 16.9)	6.2 (4.2, 8.4)	**−**8.4 (−14.1, −2.5)	**−**8.5 (−9.9, −7.0)	−0.2 (−4.7, 4.3)
β-Cryptoxanthin, μg/L	38 (23, 59)	12 (7, 22)	**−**28.8 (−39.6, −18.1)	**−**38.3 (−46.3, −30.3)	−9.5 (−23.2, 4.2)
α-Tocopherol,^3^ mg/L	5.9 (4.4, 7.6)	4.0 (2.9, 5.6)	**−**1.5 (−2.3, −0.6)	**−**2.2 (−2.8, −1.6)	−0.7 (−1.7, 0.3)
α-Tocopherol,^3^ mg/g milk fat	0.11 (0.09, 0.14)	0.10 (0.07, 0.13)	−0.02 (−0.03, 0)	**−**0.01 (−0.02, −0.01)	0.00 (−0.02, 0.02)
γ-Tocopherol, mg/L	0.51 (0.34, 0.74)	0.34 (0.24, 0.51)	−0.08 (−0.19, 0.04)	**−**0.19 (−0.26, −0.13)	−0.11 (−0.24, 0.01)
γ-Tocopherol,^3^ mg/g milk fat	0.01 (0.01, 0.01)	0.01 (0.01, 0.01)	0 (0, 0)	0 (0, 0)	−0.001 (−0.004, 0.001)
Vitamin E (as TE),^4^ mg/L	6.1 (4.5, 7.8)	4.1 (3.0, 5.7)	**−**1.5 (−2.3, −0.6)	−2.2 (−2.8, −1.6)	−0.7 (−1.8, 0.3)
Vitamin E (as TE),^3,4^ mg/g milk fat	0.12 (0.09, 0.15)	0.10 (0.07, 0.14)	−0.02 (−0.03, 0)	−0.02 (−0.02, −0.01)	0.00 (−0.02, 0.02)
Free thiamin, μg/L	17.4 (12.2, 23.4)	18.5 (13.9, 27.1)	3 (0.4, 5)	3 (1, 6)	1 (−3, 4)
TMP, μg/L	96 (80, 113)	96 (72, 110)	−4 (−12, 4)	−2 (−7, 3)	2 (−7, 12)
TPP, μg/L	4.1 (2.9, 5.7)	4.0 (2.9, 5.8)	0.4 (−0.6, 1.4)	−0.3 (−0.9, 0.3)	−0.7 (−1.8, 0.4)
Total thiamin (B-1),^5^ μg/L	104 (91, 120)	105 (89, 124)	−1 (−8, 6)	2 (−3, 6)	2 (−6, 10)
Free riboflavin, μg/L	11.9 (8.4, 16.1)	9.3 (5.8, 15.7)	−4 (−6, −1)	2 (−3, 7)	6 (−2, 13)
FAD, μg/L	163 (123, 208)	168 (124, 244)	45 (−7, 96)	19 (−2, 39)	−26 (−72, 20)
FMN, μg/L	12.9 (7.6, 24.0)	14.9 (8.8, 23.6)	2 (−1, 6)	0 (−2, 3)	−2 (−5, 2)
Total riboflavin (B-2),^6^ μg/L	106 (84, 127)	110 (82, 153)	19 (−5, 44)	11 (−0.3. 23)	−8 (−32, 15)
Nicotinamide, μg/L	329 (229, 493)	197 (127, 290)	−173 (−283, −63)	**−**175 (−222, −128)	−2 (−103, 99)
NAD, μg/L	3041 (1678, 4909)	1548 (1114, 2392)	**−**1670 (−2897, −443)	−1967 (−2633, −1302)	−298 (−1572, 976)
Total niacin (B-3),^7^ μg/L	901 (620, 1537)	497 (377, 689)	**−**480 (−731, −229)	−537 (−680, −399)	−57 (−325, 212)
Pantothenic acid (B-5), μg/L	1425 (1152, 1774)	1149 (922, 1427)	−215 (−351, −79)	−322 (−410, −233)	−107 (−265, 51)
Pyridoxal, μg/L	88 (64, 116)	101 (71, 128)	6 (−7, 18)	13 (−5, 31)	8 (−20, 35)
Pyridoxine,^8^ μg/L	0.32 (0.06, 0.37)	0.11 (0.05, 0.14)	−0.1 (−0.2, −0.1)	−0.1 (−0.3, 0.1)	0.0 (−0.2, 0.3)
Vitamin B-6,^9^ μg/L	88 (65, 116)	101 (71, 128)	5 (−8, 18)	13 (−5, 31)	8 (−20, 35)
Biotin (B-7), μg/L	7.9 (6.0, 9.5)	4.7 (2.9, 6.9)	−2.8 (−3.6, −1.9)	−2.2 (−2.9, −1.6)	0.5 (−0.6, 1.6)
Vitamin B-12, μg/L	0.29 (0.24, 0.36)	0.28 (0.24, 0.35)	0.02 (−0.07, 0.11)	−0.02 (−0.06, 0.01)	−0.05 (−0.13, 0.03)

1 MN, micronutrient; TE, tocopherol equivalent; TMP, thiamin monophosphate; TPP, thiamin pyrophosphate.

2 Mean difference (95% CI) determined using change values between 2 and 5 mo postpartum.

3 *n* = 1 missing milk fat associated MN value at 2 mo; *n* = 1 missing α-tocopherol, *n* = 1 missing milk fat associated MN value at 5 mo.

4 Vitamin E calculated as the sum of each vitamer expressed as TEs, where 1 mg α-tocopherol = 1 TE, 0.25 mg γ-tocopherol = 1 TE (10).

5 Vitamin B-1 was calculated based on the measured concentrations for each vitamer and expressed as free thiamin [thiamin + (TPP × 0.707) + (TMP × 0.871)].

6 Vitamin B-2 was calculated based on the measured concentrations for each vitamer and expressed as riboflavin [riboflavin + (FAD × 0.479) + (FMN × 0.825)].

7 Vitamin B-3 was calculated as nicotinamide [nicotinamide + (NAD × 0.184)].

8 Pyridoxine was dichotomized [due to 36.3% (*n* = 70) & 94.3% (*n* = 182) of milk concentrations at 0 μg/L at 2 and 5 mo postpartum, respectively] and reported values are based on those with pyridoxine concentrations > 0 µg/L (*n* = 124 and *n* = 11).

9 Vitamin B-6 was calculated as (pyridoxal + pyridoxine).

**TABLE 5:** Maternal biomarkers and human milk MN concentrations at 5 mo postpartum (*n* = 163)^1^

Maternal blood biomarker	Micronutrients in human milk	Mean % difference (95% CI) in human milk concentration for each unit higher biomarker^2^
Hemoglobin, g/L	Iron	1.9 (1.2, 2.6)
Iron sTfR, mg/L	Iron	−7.0 (−10.1, −3.8)
Iron BIS, mg/kg	Iron	4.6 (2.5, 6.8)
Iron serum ferritin, μg/L	Iron	0.4 (0.1, 0.7)
Zinc, μmol/L	Zinc	−3.8 (−9.7, 2.4)
RBP, μmol/L	Retinol	35.3 (10.7, 65.4)
RBP, μmol/L	β-Carotene	20.4 (−13.3, 67.3)
RBP, μmol/L	α-Carotene	19.0 (−13.2, 63.2)
RBP, μmol/L	β-Cryptoxanthin	40.3 (2.9, 91.2)
Selenium, μmol/L	Selenium	70.4 (16.0, 150.5)
Vitamin B-12, pmol/L	Vitamin B-12	0.1 (0.1, 0.2)

1 AGP, α-1-acid-glycoprotein; BIS, body iron stores; RBP, retinol binding protein; sTfR, serum transferrin receptor.

2 Regressions were run with the log-transform of the milk concentration as the outcome and mean differences (95% CI) were back-transformed and reported as % differences. Associations were adjusted for parity, milk volume, rural living status and inflammation (AGP).

Mammary zinc secretion in human milk is said to be independent of maternal diet and circulating zinc concentrations for women with adequate zinc status (60–62). Unexpectedly, in the present study the direction of the estimates between human milk zinc and both maternal zinc intake at 2 mo postpartum and status at 5 mo postpartum were negative, a trend that may be associated with the apparent suboptimal zinc status of the disadvantaged Indonesian women studied here. Several other investigators have reported a comparable inverse relation among women of low socioeconomic status (55–57). Strict precautions were employed here and in the earlier studies in the collection and analyses of the milk samples, so contamination is unlikely to be the explanation. Two-thirds of the Indonesian mothers had seemingly low adjusted serum zinc concentrations at 5 mo postpartum. However, interpretation of the low serum zinc concentrations is difficult because the cutoffs applied, although they were for morning/nonfasting serum samples (i.e., 10.7 μmol/L) (22), were not developed for lactating women, whose serum zinc concentrations may be lower (63). More studies employing rigorous methodology are needed to examine the relation between human milk zinc concentrations and maternal zinc intakes and status among women of low socioeconomic status.

### Comparison of human milk MN concentrations with EFSA accepted values

Currently, assessment of human milk quality is hindered by the absence of reference values for well-nourished, unsupplemented mothers of term infants <6 mo of age (64). A general comparison here with EFSA-accepted values for mature milk collected at unspecific infant age revealed lower concentrations for those milk B vitamins, selenium, and retinol assumed to be reduced by maternal deficits, as well as for some said to be unaffected (i.e., sodium, copper, and iron) (7) (**Table 3**). In our study participants, low milk B vitamins and retinol concentrations at 5 mo postpartum were most likely attributable to the inadequacies in maternal intakes, also seen in our previous study (12). Certainly, the maternal status of RBP at 5 mo postpartum in the present study paralleled shortfalls in vitamin A intakes at this time, and the marked difference between human milk retinol concentrations at 2 and 5 mo postpartum arose because the mothers received early postpartum supplementation (40 d after delivery) with high-dose vitamin A capsules.

Our findings of low concentrations of sodium, copper, and iron at 5 mo postpartum were unexpected, given that they are assumed to be unaffected by maternal diet or status (52, 65). Unfortunately, neither maternal intakes nor the biomarker status for sodium and copper were measured. However, breastfeeding frequency (66), as noted earlier (12), coupled with the use of a mechanical pump for human milk expression (67) may account in part for our low milk sodium concentrations. Reasons for the low milk copper concentrations are uncertain. Inadequacies in copper intakes are unlikely because copper is widely distributed in food (68). Human milk copper concentrations may have been impacted by low intakes and status of selenium among Sumedang district mothers arising from low amounts of soil selenium in this rural district, both reported earlier (12), and in the current study (mean ± SD serum selenium of urban mothers 1.17 ± 0.14 compared with that of rural mothers, 1.02 ± 0.12; *P* < 0.001) (69). Reasons for low human milk iron concentrations have been discussed earlier.

Interestingly, even though concentrations of all human milk MNs measured at 5 mo postpartum in the present study were low in relation to the EFSA-accepted values for mature milk; only the values for milk thiamin and riboflavin were associated with maternal or infant deficiencies compared with the literature (64), and very few mothers (48.7%) had human milk retinol values below the WHO cutoffs (i.e., ≤300 μg/L, ≤1.05 μmol/L, or ≤8 μg/g milk fat) at 5 mo postpartum (70).

### Human milk MN concentrations in relation to both changes between 2 and 5 mo postpartum and milk volume

On average, almost all human MN values declined between 2 and 5 mo postpartum, in accordance with the literature (7, 71–73), even though, in general, maternal intakes were unchanged. Exceptions were the suggested increases in milk concentrations of FAD, total riboflavin, and vitamin B-6 (albeit small), and for free thiamin, a mean increase of 3 μg/L over this time. In most cases, the temporal changes were independent of changes in human milk volume, except for the decline in the concentrations of milk potassium, which was significantly larger for those participants with a greater milk output.

These findings are important because reports of associations between human milk volume and changes in milk MN concentrations during the first 6 mo have not been studied in such a way before (71). Nonetheless, to our knowledge, the influence of milk volume on the concentration of milk MNs has not been studied systematically (64), making the interpretation of our findings challenging. Moreover, our lactating mothers were not adequately nourished, and thus the associations may have been confounded by their disadvantaged state.

### Advantages and limitations

Our study was longitudinal, unlike our previously reported study (12), and eliminated factors with the potential to confound relations between human milk quality and maternal status (such as diurnal variation and contamination) (74) by using standardized methods (such as collection in the morning and the use of acid-washed trace element–free glass bottles) at both 2 and 5 mo postpartum. In addition, through our DTM measurement of human milk volume we confirmed some relations between milk volume and MN quality. Nevertheless, our biomarker status of the mothers was restricted to only 4 biomarkers at 1 time point (i.e., 5 mo postpartum), including only 1 for the B-vitamins (serum vitamin B-12), despite persistent inadequacies in several other maternal B-vitamin intakes. Furthermore, because our models included measurements for each of the many variables, we excluded 9 participants initially enrolled, because of lack of data for maternal dietary intakes, biomarkers, and/or human milk samples, along with an additional 49 mothers who were missing status data and/or human milk MN concentration data. While we have assessed a large number of variables, we have focused on estimates, not statistical hypothesis tests. Therefore, we have not made any adjustment for multiplicity and this should be considered by the reader. Finally, we acknowledge that serum zinc is insensitive to changes in dietary zinc intakes so future studies should also include key metabolic indicators of zinc status along with serum and dietary zinc (75). Promising examples include DNA damage along with zinc sensitive serum proteins involved in DNA damage, such as peroxiredoxin-1, ferritin, fibrinogen, and resistin (76), and the ratios of linolenic acid:dihomo-ƴ-linolenic acid in plasma, proxy markers for Δ-6-desaturase activity (77, 78). These emerging biomarkers appear sensitive to small changes in dietary zinc intake and warrant further study.

In conclusion, among these disadvantaged Indonesian women with evidence of multiple MN inadequacies, human milk concentrations of all the B-vitamins (except biotin), retinol, sodium, copper, and iron were lower than EFSA values, with those for vitamin B-12, retinol, β-cryptoxanthin, selenium, and iron (but not zinc) being positively associated with maternal nutritional status at 5 mo postpartum. Most milk MN concentrations declined during lactation and were not affected substantially by the amount of milk produced (except for β-carotene, α-carotene, and β-cryptoxanthin). Given the few associations between maternal MN intakes and corresponding milk concentrations, further work is needed to explore whether these changes in milk MN concentrations are linked to shifts in maternal MN status in the first 6 mo postpartum. Likewise, future studies to improve maternal MN status during lactation and evaluate the impact on human milk MN concentrations in at-risk mothers are urgently required.

## Supplementary Material

nqaa200_Supplemental_Files

## Notes

Supported by the Bill & Melinda Gates Foundation, Seattle, WA, USA.

Supplemental Table 1 and Supplemental Figure 1 are available from the “Supplementary data” link in the online posting of the article and from the same link in the online table of contents at https://academic.oup.com/ajcn/.

Abbreviations used: AGP, α-1-acid-glycoprotein; AI, adequate intake; BIS, body iron stores; CRP, C-reactive protein; DTM, deuterium oxide dose-to-mother; EAR, estimated average requirement; EBF, exclusively breastfed; EFSA, European Food Safety Authority; ICP-MS, inductively coupled plasma mass spectrometry; IOM, Institute of Medicine; IZiNCG, International Zinc Nutrition Consultative Group; MN, micronutrient; PBF, partially breastfed; PL, pyridoxal; PN, pyridoxine; RBP, retinol binding protein; sTfR, serum transferrin receptor; TE, tocopherol equivalents; TMP, thiamin monophosphate; TPP, thiamin pyrophosphate; UPLC, ultra-performance liquid chromatography.
