# Supplementary material for: Association of maternal diet, micronutrient status, and milk volume with milk micronutrient concentrations in Indonesian mothers at 2 and 5 months postpartum
Source: Am J Clin Nutr. 2020 Aug 25;112(4):1039–50. doi: 10.1093/ajcn/nqaa200 (PMC7528569; doi:10.1093/ajcn/nqaa200)
Supplement: nqaa200_Supplemental_Files [file nqaa200_supplemental_files.zip › Online Supplementary Figures_10dec19.docx]

Enrolled (*n* = 221)

Excluded (*n* = 9)

- Missing biomarkers, dietary intake and milk concentrations (*n* = 9)

Included in analysis (*n* = 212)

Analysis for maternal intake (*n* = 210)

- Missing dietary intake at either 2 or 5 mo (*n* = 2)

Analysis for milk concentrations (*n* = 193)

- Missing milk data at either 2 or 5 mo (*n* = 2)

Analysis for biomarkers (*n* = 163)

- Missing biomarker data and/or milk data (*n* = 49)

**Supplementary Figure 1.** Participant flowchart.
