# Supplementary material for: Association of maternal diet, micronutrient status, and milk volume with milk micronutrient concentrations in Indonesian mothers at 2 and 5 months postpartum
Source: Am J Clin Nutr. 2020 Aug 25;112(4):1039–50. doi: 10.1093/ajcn/nqaa200 (PMC7528569; doi:10.1093/ajcn/nqaa200)
Supplement: nqaa200_Supplemental_Files [file nqaa200_supplemental_files.zip › Online Supplementary Table1.docx]

**Supplementary Table 1**

Maternal intakes as predictors of human milk micronutrient concentrations (n=207 at 2 mo, n=199 at 5 mo)^1^

| Micronutrient intakes | Micronutrients concentration in human milk | 2 mo mean % difference (95% CI) in human milk concentration for each unit greater intake^2^ | 5 mo mean % difference (95% CI) in human milk concentration for each unit greater intake^2^ |
| --- | --- | --- | --- |
| Calcium, mg/d | Calcium | **0.01 (0, 0.03)** | -0.01 (-0.02, 0.01) |
| Potassium, g/d | Potassium | -4.1 (-11.5, 3.3) | 0.2 (-6.3, 6.7) |
| Iron, mg/d | Iron | **2.4 (0.3, 4.5)** | 0.9 (-1.1, 3.0) |
| Zinc, mg/d | Zinc | **-3.9 (-6.7, -1.0)** | -2.7 (-5.5, 0.1) |
| Vitamin A, μg RAE/d | Retinol | 0.01 (-0.01, 0.04) | 0.01 (-0.01, 0.06) |
| Vitamin A, μg RAE/d | Retinol, milk fat | 0 (-0.02, 0.03) | 0.02 (-0.04, 0.07) |
| Vitamin A, μg RAE/d | β-Carotene | 0.03 (-0.01, 0.07) | **0.08 (0.02, 0.13)** |
| Vitamin A, μg RAE/d | α-Carotene | 0.01 (-0.03, 0.04) | 0.02 (-0.04, 0.07) |
| Vitamin A, μg RAE/d | β-Cryptoxanthin | 0.02 (-0.02, 0.06) | 0.05 (-0.01, 0.10) |
| Thiamin (B-1), mg/d | Free thiamin | 5.6 (-17.5, 28.7) | -0.9 (-19.6, 17.7) |
| Thiamin (B-1), mg/d | TMP | 15.7 (-9.4, 40.8) | -4.7 (-28.2, 18.7) |
| Thiamin (B-1), mg/d | TPP | 10.3 (-46.5, 37.1) | -10.4 (-28.3, 7.6) |
| Thiamin (B-1), mg/d | Total thiamin (B-1)^3^ | 9.1 (-5.7, 23.9) | -3.4 (-15.8, 9.0) |
| Riboflavin (B-2), mg/d | Free riboflavin | 25.2 (-4.2, 54.6) | -0.4 (-41.7, 40.9) |
| Riboflavin (B-2), mg/d | FAD | 3.7 (-20.9, 28.3) | 3.0 (-15.7, 21.8) |
| Riboflavin (B-2), mg/d | FMN | -8.2 (-46.0, 29.6) | 5.1 (-22.5, 32.6) |
| Riboflavin (B-2), mg/d | Total riboflavin (B-2)^4^ | 8.0 (-12.0, 28.0) | 5.6 (-11.3, 22.5) |
| Niacin (B-3), mg/d | Nicotinamide | **3.9 (1.6, 6.2)** | 1.5 (-0.9, 3.8) |
| Niacin (B-3), mg/d | NAD | -0.5 (-3.6, 2.5) | -0.9 (-3.2, 1.5) |
| Niacin (B-3), mg/d | Total niacin (B-3)^5^ | 1.2 (-1.3, 3.6) | 0 (-2.0, 2.0) |
| B-6, mg/d | Pyridoxal | 8.3 (-10.0, 26.6) | 10.0 (-1.3, 21.3) |
| B-6, mg/d | Vitamin B-6^6^ | 8.4 (-9.9, 26.6) | 10.1 (-1.2, 21.4) |
| B-12, μg/d | Vitamin B-12 | 1.8 (-3.9, 7.5) | 0.1 (-1.3, 1.5) |

^1^ **Bold**: Indicates *P* < 0.05. TMP, Thiamin monophosphate; TPP, Thiamin pyrophosphate; FAD, Flavin Adenine Dinucleotide; FMN, Flavin Mononucleotide; NAD, Nicotinamide Adenine Dinucleotide; RAE, retinol activity equivalents.

^2^ Regression models with log-transformed human milk concentration for each nutrient as the outcome with associations presented as percentage differences; models adjusted for parity and human milk volume.

^3^ Vitamin B-1 was calculated based on the measured concentrations for each vitamer and expressed as free thiamin [thiamin + (TPP × 0.707) + (TMP × 0.871)].

^4^ Vitamin B-2 was calculated based on the measured concentrations for each vitamer and expressed as riboflavin [riboflavin + (FAD × 0.479) + (FMN × 0.825)].

^5^ Vitamin B-3 was calculated as nicotinamide [nicotinamide + (NAD × 0.184)].

^6^ Vitamin B-6 was calculated as (pyridoxal + pyridoxine).
